# Cross-Cultural Adaptation and Validation of the Arabic Rating-of-Fatigue Scale

**DOI:** 10.1186/s40798-026-01052-7

**Published:** 2026-06-19

**Authors:** Mohamed Ali Baccouche, Khaled Trabelsi, Liwa Masmoudi, Haitham Jahrami, Achraf Ammar, Hamdi Chtourou

**Affiliations:** 1 High Institute of Sport and Physical Education, University of Sfax, Sfax, Tunisia; 2 Research Laboratory, Education, Motricity, Sport and Health (EM2S), LR15JS01, High Institute of Sport and Physical Education, University of Sfax, Sfax, Tunisia; 3 Department of Movement Sciences and Sports Training, School of Sport Science, The University of Jordan, Amman, Jordan; 4 Department of Psychiatry, Ministry of Health, Manama, Bahrain; 5 Department of Psychiatry, College of Medicine and Medical Sciences, Arabian Gulf University, Manama, Bahrain; 6 Research Laboratory, Molecular Bases of Human Pathology, LR19ES13, Faculty of Medicine of Sfax, University of Sfax, 3000 Sfax, Tunisia; 7 Department of Training and Movement Science, Institute of Sport Science, Johannes Gutenberg-University Mainz, Mainz, Germany; 8 Department of Nutrition and Food Technology, School of Agriculture, The University of Jordan, Amman, Jordan; 9 Research Unit, Physical Activity, Sport, and Health, UR18JS01, National Observatory of Sport, 1003 Tunis, Tunisia

**Keywords:** Perceived fatigue, Exertion scale, Heart rate, Construct validity, Face validity, Cross-cultural adaptation

## Abstract

**Background:**

The Rating-of-Fatigue (ROF) scale is increasingly used to quantify subjective sensations of fatigue in exercise science. However, despite its growing international use, its psychometric properties have not yet been examined in Arabic-speaking populations. This study aimed to translate the ROF into Arabic and evaluate its content validity, face validity, and construct validity relative to perceived exertion and heart rate (HR) responses during graded exercise and subsequent passive recovery.

**Methods:**

The study was conducted in Tunisia and followed established international best-practice guidelines for cross-cultural adaptation. The ROF and its instructions were forward-translated, synthesized, and back-translated using blinded translators, with linguistic and conceptual equivalence evaluated using Sperber’s framework and expert committee review. Face validity was assessed in 68 Arabic-speaking adults. Construct validity was examined in 43 adult participants during a modified Bruce treadmill test followed by 10 min of seated recovery, with the Arabic Rating-of-Fatigue scale (ROF-Ar), Borg CR10, and HR recorded at each exercise stage and during recovery. Within-participant correlations were pooled using Fisher’s r-to-z transformation.

**Results:**

Sperber-based ratings indicated high linguistic and conceptual equivalence between the ROF-Ar and the original scale, with no item exceeding the predefined revision threshold. Face validity findings showed that participants predominantly interpreted the ROF-Ar as a measure of fatigue rather than exertion and that standardized instructions improved descriptor-related comprehensibility and reduced perceived difficulty. During graded exercise, ROF-Ar demonstrated very strong pooled correlations with Borg CR10 (*r* = 0.94; 95% CI 0.92–0.95; *p* < 0.001) and HR (*r* = 0.95; 95% CI 0.93–0.96; *p* < 0.001). During recovery, the association between ROF-Ar and Borg CR10 attenuated substantially (*r* = 0.35; 95% CI 0.15–0.52; *p* < 0.001), whereas the association with HR remained moderate (*r* = 0.63; 95% CI 0.49–0.74; *p* < 0.001).

**Conclusions:**

The Arabic version of the ROF demonstrated high linguistic and conceptual equivalence, adequate face validity, and strong construct validity. The recovery-phase dissociation between perceived fatigue and perceived exertion supports the intended conceptual distinction of the ROF and justifies the use of ROF-Ar for assessing subjective fatigue during exercise and recovery in Arabic-speaking research and applied settings.

**Supplementary Information:**

The online version contains supplementary material available at 10.1186/s40798-026-01052-7.

## Key Points

- The Arabic Rating-of-Fatigue scale (ROF-Ar) demonstrated strong linguistic, conceptual, and face validity in Arabic-speaking adults.
- The ROF-Ar was closely associated with perceived exertion during exercise but diverged from it during recovery, supporting its construct validity and discriminant validity.
- The ROF-Ar provides information on postexercise fatigue that is not fully captured by heart rate or exertion-based measures, thereby improving fatigue assessment in Arabic-speaking research and applied settings.

**Supplementary Information:**

The online version contains supplementary material available at 10.1186/s40798-026-01052-7.

## Introduction

Fatigue is a multidimensional phenomenon that has attracted interest across several fields, including exercise physiology, neuroscience, and health psychology. However, it remains difficult to define precisely because of its complexity, the heterogeneity of its manifestations, and the variety of its underlying origins reported in the literature [1–4]. This conceptual heterogeneity has been identified as a barrier to theoretical coherence, potentially limiting the translation of research findings into effective performance optimization and clinical interventions [1, 2].

To address these differing perspectives, an influential conceptual framework distinguishes between two related components of fatigue. Performance fatigability refers to the objectively measurable decline in force or power output during exercise, whereas perceived fatigability captures the subjective experience of fatigue as internal states evolve [1, 2]. Considered together, these dimensions provide a broader understanding of fatigue by linking observable changes in performance to the individual’s perceptual experience. This distinction also has important implications for measurement, as fatigue cannot be fully characterized using physiological outcomes alone.

From a measurement perspective, this distinction is particularly relevant because internal load during exercise is often monitored using heart rate (HR) and perceived exertion, such as the Borg Category-Ratio 10 scale (Borg CR10) [5–7]. Although these indicators are useful, they primarily reflect the intensity of effort rather than the subjective experience of fatigue itself [1, 8, 9]. This limitation is especially evident during recovery, when physiological strain and perceived exertion may decline rapidly while the sensation of fatigue persists [1, 8, 9]. Accordingly, physiological and exertion-based indicators alone may not fully capture the exercise–recovery continuum.

In this context, Micklewright and colleagues developed the Rating-of-Fatigue (ROF) scale, a simple 0–10 perceptual scale designed to quantify perceived fatigue “here and now” across both exercise and recovery contexts [8]. The ROF integrates physical and mental dimensions of fatigue into a single rating and was explicitly developed to assess perceived fatigue rather than perceived exertion [8]. Initial validation of the original ROF demonstrated strong construct validity, with close tracking of physiological and perceptual markers during graded exercise and clear dissociation from perceived exertion during recovery [8]. Validated adaptations are also available in French [10] and Portuguese [11], supporting the international relevance of the instrument. However, these adaptations do not guarantee equivalence across other linguistic and cultural contexts, particularly for perceptual instruments, where content validity depends on the accurate interpretation of anchors, descriptors, and instructions within the target language.

For an instrument to be used internationally, literal translation is insufficient. Cross-cultural adaptation is required to ensure semantic, conceptual, and operational equivalence, facilitating consistent interpretation of scale anchors, descriptors, and instructions across populations [12]. Accordingly, Beaton’s guidelines and the COSMIN (Consensus-Based Standards for the Selection of Health Measurement Instruments) framework recommend a structured process involving independent forward translations, synthesis, blinded back-translation, expert committee review, and subsequent evaluation of validity and reliability in the target population [12–14]. The aim is to ensure that the translated version conveys the same meaning and guides scoring in the same way as the original instrument, thereby minimizing interpretation errors [12–14].

Despite the growing international use of the ROF, no standardized and validated Arabic version has been available to date, which limits its applicability in Arabic-speaking contexts. Adapting the scale into Modern Standard Arabic (MSA), widely used across educational and official communication settings, may support broader use across Arabic-speaking populations. Because the ROF is a perceptual instrument, Arabic validation is required to determine whether the translated version preserves the intended meaning of the original scale and continues to distinguish perceived fatigue from related constructs, particularly perceived exertion [7, 8, 12–14]. This issue is especially relevant in settings such as training monitoring, rehabilitation, functional assessment, and recovery evaluation, where a specific measure of perceived fatigue can complement physiological markers and exertion scales and thereby inform interpretation and decision-making in real time [7, 8].

Therefore, the present study aimed to translate, cross-culturally adapt, and validate the ROF in MSA (ROF-Ar) in accordance with Beaton’s guidelines and the COSMIN framework [12–14]. Specifically, the objectives were to (i) ensure linguistic and conceptual equivalence of the Arabic version, including its anchors, descriptors, and instructions; (ii) evaluate face validity in Arabic-speaking adults and (iii) examine construct validity, operationalized through convergent validity during exercise and discriminant validity during recovery, using a standardized exercise–recovery protocol.

## Methods

### Study Design and Validation Framework

This study followed a structured, multiphase validation framework to translate, cross-culturally adapt, and evaluate the psychometric properties of the ROF in MSA. The methodological approach was informed by established international recommendations for the cross-cultural adaptation of self-reported outcome measures, as well as by contemporary validation frameworks, including principles outlined by Beaton and colleagues and the COSMIN initiative [12–16].

The empirical validation procedures were conducted in Sfax, Tunisia, and comprised three sequential phases:

(i) Translation and cross-cultural adaptation of the ROF,
(ii) Assessment of face validity in Arabic-speaking adults, and
(iii) Evaluation of construct validity through convergent and discriminant analyses during graded exercise and post-exercise recovery.

**Fig. 1:**
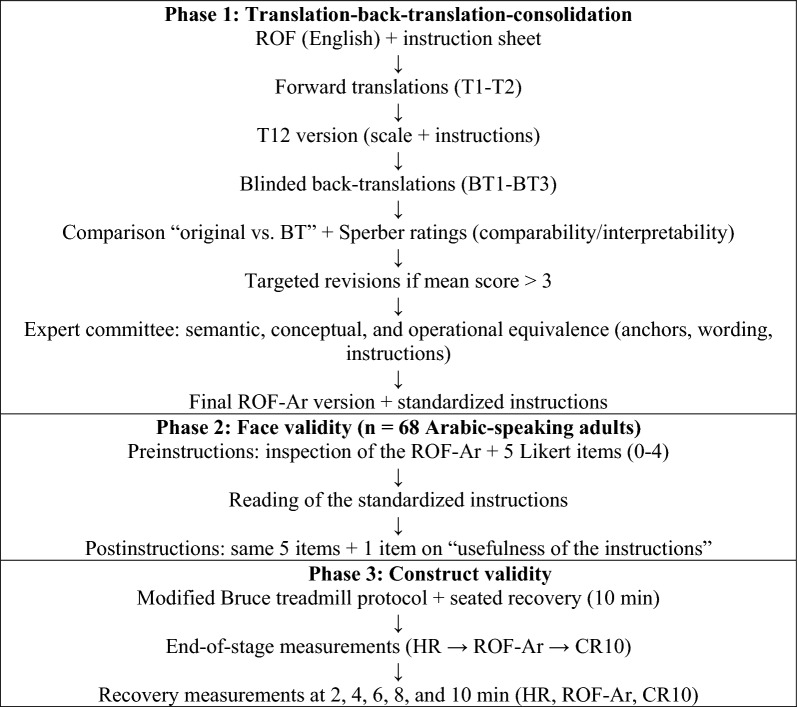
Translation, cross-cultural adaptation, and validation process for the ROF-Ar. Abbreviations: ROF, Rating-of-Fatigue Scale; ROF-Ar, Arabic version of the Rating-of-Fatigue scale; T1/T2 = forward translations; T12 = synthesized version; BT = back-translations; HR = heart rate; CR10 = Borg CR10

An overview of the study design is presented in Fig. 1.

### Ethical Considerations

Ethical approval was obtained from the Institutional Review Board at the High Institute of Sport and Physical Education of Sfax (ISSEP/019), University of Sfax, Tunisia. The study was conducted in accordance with the ethical principles of the Declaration of Helsinki and its later amendments. All participants received written and verbal information about the study procedures and provided written informed consent prior to participation. The study protocol was not prospectively registered in a public database.

### Sample Size Justification

For face validity, sample size was informed by previous ROF validation studies, which generally included approximately 60–73 participants [8, 10, 11]. Accordingly, the phase 2 sample (*n* = 68) was considered appropriate for an initial face-validity assessment.

For construct validity, sample size was determined a priori using recovery-phase effect sizes reported in previous ROF studies and cross-cultural validations [8, 10, 11]. Power calculations based on Fisher’s z transformation indicated that detecting a moderate association (*r* ≈ 0.45) with *α* = 0.05 (two-tailed) and 80% power required approximately 35–40 participants. Accordingly, a target sample of 40–50 participants was considered appropriate for phase 3, and 43 participants were included.

### Phase 1: Translation and Cross-Cultural Adaptation

Two independent native Arabic-speaking translators, both fluent in English, translated the ROF and its instruction sheet from English into MSA. One translator was familiar with sport and biomedical terminology, whereas the other had no specific background in the construct under study, thereby providing complementary perspectives.

The two forward translations were reconciled through a consensus process involving the translators and a coordinating member of the research team, who reviewed discrepancies in wording, meaning, and register before agreeing on the synthesized Arabic version (T12). This version was subsequently back-translated into English by two independent native English-speaking translators, all blinded to the study objectives and without access to the original ROF materials. This blinding procedure was implemented to minimize confirmation bias and enhance the detection of semantic or conceptual discrepancies. Equivalence between the original version and the back-translations was assessed using Sperber’s Comparability/Interpretability Rating Sheet (1–7 scale) [17] by a panel of four independent raters, including at least two native English speakers. The raters had complementary expertise in exercise science, bilingual translation, and/or measurement validation. A mean score greater than 3 on either dimension was considered indicative of inadequate equivalence and prompted targeted revision. An expert committee composed of bilingual specialists in exercise science, psychometrics, and language translation reviewed the original ROF, forward translations, synthesized version, back-translations, and rater comments to evaluate semantic, conceptual, cultural, and operational equivalence. Discrepancies were resolved through consensus, and wording was refined when necessary to preserve the intended meaning of the original anchors, descriptors, and instructions. Following this process, the final ROF-Ar version, including the scale and standardized instructions, was established.

### Phase 2: Face Validity

#### Participants

Face validity was assessed in 68 Arabic-speaking adults (31 women and 37 men; mean age, 38.2 ± 10.8 years). Recruitment and assessment were conducted between May and August 2025 using purposive and convenience sampling. Demographic and anthropometric characteristics, together with the sex-stratified distribution of professional backgrounds, are presented in Table 1.

**Table 1:** Participant characteristics in phase 2 (mean ± SD)

Parameter	Female (*n* = 31)	Male (*n* = 37)	All (*n* = 68)
Sex, *n* (%)	31 (45.6)	37 (54.4)	68 (100)
Age (years)	40.7 ± 11.3	36.1 ± 10.2	38.2 ± 10.8
Height (cm)	159.7 ± 6.8	172.9 ± 6.3	167.1 ± 9.3
Body mass (kg)	69.4 ± 12.3	79.6 ± 11.7	74.9 ± 12.6
BMI (kg·m⁻^2^)	27.2 ± 4.8	26.6 ± 4.2	26.9 ± 4.5
Resting HR (bpm)	74.8 ± 6.1	72.5 ± 7.0	73.6 ± 6.7
*Professional background, n (%)*			
Sport and exercise science academics	5 (16.1)	9 (24.3)	14 (20.6)
Physical education teachers	6 (19.4)	8 (21.6)	14 (20.6)
Club-based athletes	10 (32.3)	4 (10.8)	14 (20.6)
Academics from nonsport disciplines	3 (9.7)	5 (13.5)	8 (11.8)
Physiotherapists	2 (6.5)	2 (5.4)	4 (5.9)
Strength and conditioning coaches/personal trainers	1 (3.2)	3 (8.1)	4 (5.9)
Sports physicians	1 (3.2)	3 (8.1)	4 (5.9)
Other physical activity professionals	2 (6.5)	2 (5.4)	4 (5.9)
Healthcare staff	1 (3.2)	1 (2.7)	2 (2.9)

BMI, body mass index; bpm, beats per minute; HR, heart rate; SD, standard deviation. Values are presented as mean ± SD unless otherwise indicated. Resting HR was measured under standardized seated resting conditions

The sampling strategy aimed to ensure heterogeneity in professional profiles and familiarity with physical activity, thereby enhancing the transferability of findings across diverse use contexts. Recruitment targeted academic, educational, sport, exercise, and health-related settings to include adults with varied professional backgrounds and different levels of familiarity with physical activity and perceptual monitoring tools.

Eligibility criteria included being aged 18 years or older, Arabic-speaking, proficient in MSA, able to read and understand the Arabic version of the scale and its instructions, and able to provide written informed consent. Exclusion criteria were inability to read or understand the Arabic instructions, cognitive or communication difficulties that could interfere with completion of the face-validity assessment, or prior involvement in the translation, back-translation, expert-review, or committee stages of the study.

#### Procedure

The participants first inspected the ROF-Ar without reading the instruction sheet and completed five Likert-scale items (0 = strongly disagree to 4 = strongly agree) assessing whether the scale (i) represented fatigue, (ii) represented exertion, (iii) was easy to understand based on its descriptors, (iv) helped assign a rating, and (v) was difficult to understand (reverse-worded). Participants then read standardized instructions emphasizing the assessment of fatigue “here and now” and the conceptual distinction between fatigue and exertion. The same five items were subsequently completed again, together with an additional item assessing the perceived usefulness of the instructions.

### Phase 3: Construct Validity

#### Participants

Construct validity was examined in 43 Arabic-speaking adults (21 women and 22 men; mean age, 35.0 ± 7.8 years). Participants were recruited and tested between September and December 2025 using convenience sampling from the university and local community, including academic, sport, and exercise-related networks. Eligibility criteria included being aged 18 years or older, Arabic-speaking, proficient in MSA, able to provide written informed consent, and medically cleared to perform maximal treadmill exercise testing. Exclusion criteria were any known cardiovascular, respiratory, metabolic, neurological, or musculoskeletal condition contraindicating maximal exercise, current injury or acute illness that could limit treadmill running, use of medication likely to substantially affect heart-rate response to exercise, inability to comply with the testing procedures, or any safety concern identified by the test supervisor.

Based on self-reported athletic status, 17 participants (39.5%) were classified as athletes, including 8 females (38.1% of females) and 9 males (40.9% of males). The remaining 26 participants (60.5%), comprising 13 females (61.9% of females) and 13 males (59.1% of males), were classified as non-athletes. Anthropometric and physiological characteristics (height, body mass, BMI, and resting HR), together with participants’ distribution by sex, are presented in Table 2.

**Table 2:** Participant characteristics in phase 3 (mean ± SD)

Parameter	Female (*n* = 21)	Male (*n* = 22)	All (*n* = 43)
Sex, *n* (%)	21 (48.8)	22 (51.2)	43 (100)
Age (years)	35.6 ± 8.2	34.4 ± 7.6	35.0 ± 7.8
Height (cm)	162.5 ± 5.4	174.9 ± 5	168.8 ± 8.1
Body mass (kg)	64.6 ± 8.6	75.2 ± 9.9	70.0 ± 10.6
BMI (kg.m^−2^)	24.5 ± 3.1	24.6 ± 3.6	24.6 ± 3.4
Resting HR (bpm)	71.9 ± 4.0	70.9 ± 5.3	71.3 ± 4.7

BMI, body mass index; bpm, beats per minute; HR, heart rate; SD, standard deviation. Values are presented as mean ± SD unless otherwise indicated. Resting HR corresponds to seated pre-test HR measured under standardized seated conditions before the exercise protocol

Proficiency in MSA was considered essential because the target instrument was designed for Arabic-speaking populations; however, this does not imply equivalent proficiency in secondary languages such as French, even when these are used in applied contexts for more familiar or less linguistically demanding instruments such as the Borg CR10. Standardized presession instructions were provided, including abstinence from caffeine and nicotine for at least 3 h, avoidance of vigorous exercise during the preceding 24 h, and consumption of the last meal at least 2 h before testing.

#### Construct Validity Framework

Construct validity was examined through convergent and discriminant analyses. Convergent validity was evaluated by examining associations between ROF-Ar, perceived exertion (Borg CR10), and HR during graded exercise, where fatigue and exertion are expected to increase in parallel with physiological load [1, 2, 8, 9]. Discriminant validity was evaluated during the post-exercise recovery period, where perceived fatigue was expected to diverge from perceived exertion and HR, reflecting distinct perceptual responses during recovery [1, 2, 8, 9].

#### Exercise Protocol

Assessments were conducted on a motorized clinical treadmill (COSMED T170 Clinical/Pulmonary Function Treadmill, model T170 DE; COSMED Srl, Germany) using a modified Bruce protocol. After a 5-min warm-up (6 km·h⁻^1^, 0% grade), the test began at 7 km·h⁻^1^ (1% grade). The speed increased by 1 km·h⁻^1^ every 2 min up to 13 km·h⁻^1^. Speed was then maintained at 13 km·h⁻^1^ while the treadmill grade increased by 2% every 2 min until volitional exhaustion or the attainment of standardized termination criteria [18, 19]. A 10-min seated recovery period followed immediately after exercise cessation.

Exercise testing was terminated upon participant request or when one or more of the following criteria were met: inability to maintain the required treadmill speed despite encouragement, attainment of age-predicted maximal HR (± 10 beats·min⁻^1^), appearance of symptoms indicative of exercise intolerance (e.g., dizziness, chest discomfort, severe dyspnea, or nausea), or any safety concern identified by the test supervisor.

HR was recorded continuously using a validated Polar H10 monitor (≥ 1 Hz), with no visual feedback provided to participants to minimize perceptual anchoring [20].

#### Measurement Timing and Order

At the end of each exercise stage (final 15 s) and at each recovery time point, measurements were collected in a fixed order to minimize perceptual interference: (i) HR (15-s average), (ii) ROF-Ar (0–10; integer ratings), and (iii) Borg CR10 (0–10; decimal ratings permitted). During recovery, HR, ROF-Ar, and Borg CR10 were recorded at 2, 4, 6, 8, and 10 min post-exercise.

Administration was standardized using A4 posters and a scripted set of instructions. A brief familiarization period (1–2 min) reviewed the distinction between ROF-Ar and Borg CR10 [7, 8]. The data were double-entered, and calibration procedures and any testing incidents were documented to ensure traceability.

### Instruments

#### Rating-of-Fatigue Scale (ROF)

The ROF is a single-item perceptual scale ranging from 0 to 10, developed to quantify perceived fatigue “here and now” across exercise and recovery contexts [8]. A score of 0 corresponds to “not fatigued at all”, whereas a score of 10 corresponds to “total fatigue and exhaustion, with no energy remaining”. Intermediate anchor descriptors are provided at selected points on the scale, in accordance with the original ROF design, to guide respondents in selecting the integer value that best reflects their current state. The Arabic version (ROF-Ar) retained the original scale structure, anchor logic, and integer scoring format, with adaptations limited to linguistic and cultural equivalence.

#### Perceived Exertion (Borg CR10)

Perceived exertion was assessed using the French version of Borg CR10, with decimal ratings permitted [7, 21]. Participants were provided with the main verbal anchors (0 = none; 3 = moderate; 5 = strong; 7 = very strong; 10 = maximal) and could report intermediate values (e.g., 3.5). The scale was displayed on a separate laminated A4 poster placed next to the ROF-Ar to minimize confusion between the two instruments. A standardized script was read aloud: “The CR10 measures the effort you are currently exerting. You may use decimal values.” The CR10 was administered immediately after the ROF-Ar during the final 15 s of each stage. Participants responded verbally, and the assessor recorded the rating immediately.

### Statistical Analysis

All analyses were performed using IBM SPSS Statistics version 29 (IBM Corp., Armonk, NY, USA). Tests were two-tailed with *α* = 0.05. Continuous variables are reported as means ± standard deviations (SD). Distributional assumptions were screened using the Shapiro‒Wilk test, and nonparametric procedures were used when appropriate.

For phase 2 (face validity), the five Likert items (0–4) were compared before and after instruction using paired Wilcoxon signed-rank tests for paired ordinal data. The effect size was calculated as $r=\frac{Z}{\sqrt{N}}$, where N denotes the number of paired observations contributing to each test (excluding missing values and pairs with zero differences). Holm’s step-down procedure was applied to control the familywise error rate across the five pre–post comparisons, and Holm-adjusted *p* values are reported. The reverse-worded “overall difficulty” item was interpreted as indicating improved clarity when scores decreased after instruction. The “usefulness of the instructions” item, assessed only post-instruction, was summarized descriptively without inferential testing. Missing values were handled by pairwise deletion within each comparison, and tied ranks were treated according to the standard Wilcoxon procedure.

For phase 3 (construct validity), descriptive plots expressed exercise stages as a percentage of time to exhaustion (0% = start of the modified Bruce protocol; 100% = exhaustion), with recovery time points displayed on an extended scale (100–200%). HR was extracted from the final ~15 s of each exercise stage and at 2, 4, 6, 8, and 10 min of seated recovery.

Within-participant associations (ROF-Ar vs Borg CR10, ROF-Ar vs HR, and Borg CR10 vs HR) were quantified by computing Pearson correlation coefficients separately for each participant during graded exercise and during recovery. Individual correlations were Fisher r-to-z transformed and combined at the group level using inverse-variance weighting proportional to (_*ni*_ − 3), where n_i_ is the number of paired observations available for participant i in the corresponding period. Weighted mean z values were back-transformed to report pooled r values and 95% confidence intervals. Statistical significance was evaluated using a one-sample t-test of Fisher z values against 0 (two-tailed, *α* = 0.05). This approach was used to maintain methodological comparability with the original and French ROF validation studies. Given the repeated-measures structure of the data, the pooled coefficients were interpreted as descriptive summaries of within-participant associations rather than as estimates from a mixed-effects framework.

## Results

### Phase 1: Translation and Cross-Cultural Adaptation

The translation and cross-cultural adaptation process resulted in an MSA version of the ROF with high semantic, linguistic, and conceptual equivalence to the original instrument (Figure S1). Based on Sperber’s comparability and interpretability framework, all anchors and instruction items received favorable ratings, and no item exceeded the predefined revision threshold, indicating that further wording modifications were unnecessary.

Expert committee review confirmed that the ROF-Ar preserved the intended meaning, anchor structure, and operational logic of the original scale. Detailed item-level equivalence results are presented in Table 3.

**Table 3:** Sperber-based assessment of ROF-Ar comparability and interpretability

Subset	Comparability (mean ± SD)	Interpretability (mean ± SD)	Items > 3 (after iteration), n
Anchors (A0–A10)	1.40 ± 0.34	1.28 ± 0.29	0
Instructions (I1–I3)	1.27 ± 0.38	1.07 ± 0.09	0
Overall (A0–A10, I1–I3)	1.37 ± 0.35	1.24 ± 0.28	0

Mean (± SD) values were calculated from ratings provided by four evaluators, aggregated at the item level and then within each subset (A0–A10 = 11 items; I1–I3 = 3 items; overall = 14 items). ROF-Ar, Arabic version of the Rating-of-Fatigue scale; SD, standard deviation. Scale range: 1–7 (1 = extremely comparable/similar; 7 = not at all comparable/similar). Revision threshold: mean > 3

### Phase 2: Face Validity

**Fig. 2:**
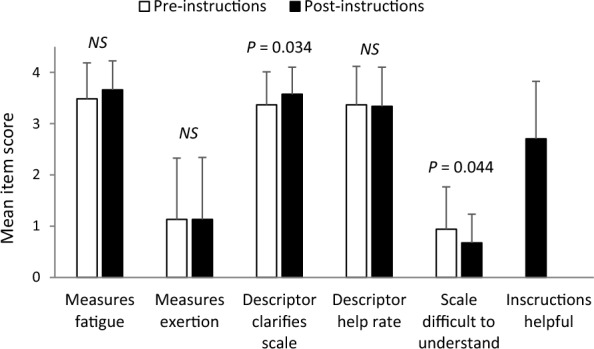
Face validity of the Arabic version of the Rating-of-Fatigue scale (ROF-Ar) before and after the instructions were read. Values are presented as mean ± SD. NS = not significant

Mean face-validity ratings obtained before and after reading the standardized instructions are presented in Fig. 2.

Participants predominantly perceived the ROF-Ar as measuring fatigue, with high scores both before and after the instructions were read (3.49 ± 0.70 vs. 3.66 ± 0.56), with no statistically significant difference after Holm adjustment (*p*Holm > 0.05).

For the item assessing whether the scale measured exertion, mean scores remained low and unchanged before and after the instructions (1.13 ± 1.20 vs. 1.13 ± 1.21). However, 19 of 68 participants (27.9%) before instructions and 22 of 68 participants (32.4%) after instructions rated this item ≥ 2/4. Because a score of 2 corresponded to “hesitant”, these proportions reflect uncertainty rather than clear agreement. Definite endorsement, defined as scores of 3–4, was observed in 14 of 68 participants (20.6%) before instructions and 10 of 68 participants (14.7%) after instructions.

Descriptor-based comprehensibility increased significantly after reading the instructions (3.37 ± 0.64 vs. 3.57 ± 0.53; *p*Holm = 0.034), whereas ratings reflecting the usefulness of descriptors for selecting a score remained high and stable (*p*Holm > 0.05).

The perceived overall difficulty of understanding the scale was low before instructions and decreased further afterward (0.94 ± 0.83 vs. 0.68 ± 0.56; *p*Holm = 0.044), indicating improved clarity. The perceived usefulness of the instruction sheet, assessed only after instructions, was 2.71 ± 1.12, reflecting moderate-to-high perceived benefit.

### Phase 3: Construct Validity

**Fig. 3:**
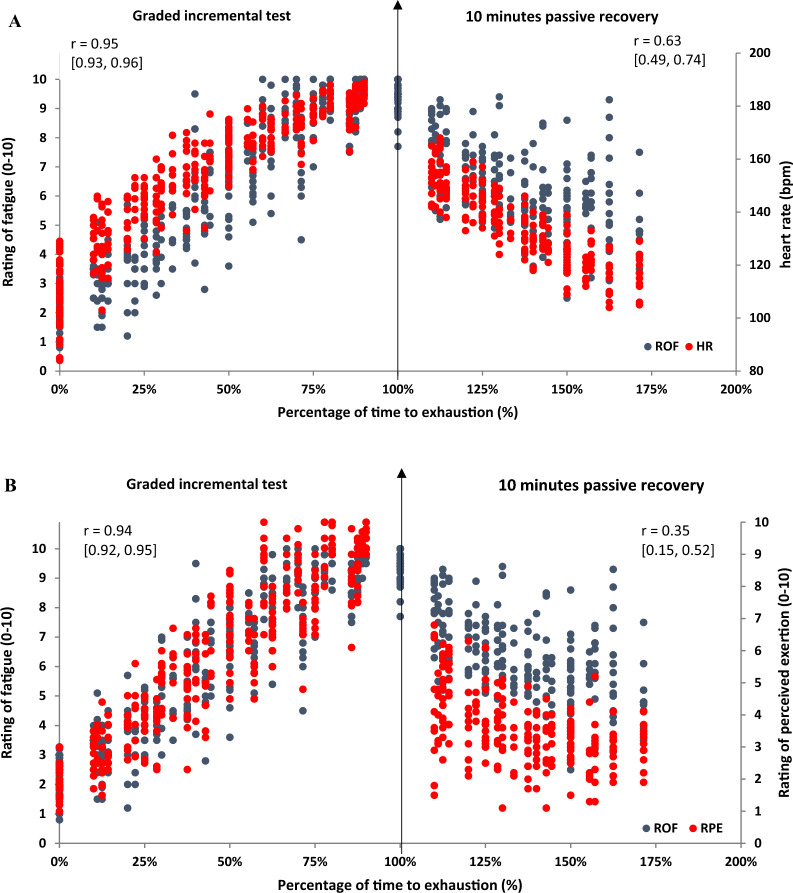
Relationship between ratings of fatigue (ROF-Ar) and HR (**A**) and between ratings of fatigue (ROF-Ar) and perceived exertion (Borg CR10) (**B**) during the graded treadmill test and 10 min of seated passive recovery. Each dot represents an individual observation

The patterns of association between ROF-Ar, perceived exertion (Borg CR10), and HR during graded exercise and recovery are presented in Table 4 and illustrated in Fig. 3.

**Table 4:** Within-participant correlations between ROF-Ar, Borg CR10, and HR during graded treadmill exercise and 10-min seated recovery

	Within-participant correlations		
	*r*	95% CI	*p* value
*Graded exercise*			
ROF-Ar vs. CR10	0.94	0.92, 0.95	< 0.001
ROF-Ar vs. HR	0.95	0.93, 0.96	< 0.001
CR10 vs. HR	0.95	0.93, 0.96	< 0.001
*10-min seated passive recovery*			
ROF-Ar vs. CR10	0.35	0.15, 0.52	< 0.001
ROF-Ar vs. HR	0.63	0.49, 0.74	< 0.001
CR10 vs. HR	0.61	0.47, 0.73	< 0.001

ROF-Ar, Arabic version of the Rating-of-Fatigue scale; CR10, Borg Category-Ratio 10 perceived exertion scale; HR, heart rate; CI, confidence interval

During the graded treadmill protocol, ROF-Ar demonstrated very strong within-participant associations with both Borg CR10 and HR. All three variables increased monotonically across exercise stages, reflecting synchronized perceptual and physiological responses to increasing workload (Fig. 3A). As expected in an incremental, stage-ordered protocol, these strong associations partly reflect shared task progression rather than construct redundancy.

During post-exercise recovery, the association between ROF-Ar and Borg CR10 weakened compared with exercise, whereas the association between ROF-Ar and HR remained moderate. Descriptively, Borg CR10 and HR declined rapidly after exercise cessation, whereas ROF-Ar decreased more gradually and showed greater interindividual variability (Fig. 3B). This pattern indicates weaker convergence between perceived fatigue and perceived exertion during recovery, consistent with the expected discriminant behavior of the ROF-Ar. The numerical values and 95% confidence intervals for these associations are reported in Table 4.

## Discussion

The aim of this study was to translate, cross-culturally adapt, and validate the ROF in Modern Standard Arabic (ROF-Ar) using a structured, multiphase methodological approach. Taken together, the present findings provide consistent evidence extending from linguistic and conceptual equivalence to face validity and construct validity assessed during both exercise and recovery. Overall, this pattern closely mirrors the psychometric profile reported in the original ROF validation and in subsequent cross-cultural adaptations, supporting the robustness and cross-linguistic consistency of the scale [8, 10, 11].

Methodologically, translation, synthesis, blinded back-translation, and expert committee review were conducted in line with foundational cross-cultural adaptation recommendations [12, 15]. Sperber-based comparability and interpretability ratings further indicate that the Arabic version conveys the original instrument’s intended meaning rather than relying on a purely word-for-word rendering [17, 22]. Comparable low equivalence scores were reported in the French and Portuguese ROF adaptations, indicating that the Arabic version achieved a level of semantic and conceptual fidelity that was consistent with previously validated versions [10, 11]. This is consistent with the COSMIN guidance, which places content validity at the core of any rigorous evaluation of measurement properties [13, 16].

Face validity findings indicate that participants predominantly interpreted the ROF-Ar as a measure of fatigue rather than exertion. Reading the standardized instruction sheet improved descriptor-related comprehensibility and reduced perceived difficulty while leaving the overall interpretation of the construct unchanged [8, 10, 11]. This pattern closely aligns with findings from the original ROF study and from the French and Portuguese validations, in which instructions enhanced clarity without altering construct interpretation [8, 10, 11]. Together, these convergent results suggest that the conceptual distinction between perceived fatigue and perceived exertion is preserved across languages when standardized instructions are provided. However, the proportion of participants showing hesitation or partial endorsement for the exertion item suggests that the fatigue–exertion distinction may still benefit from explicit explanation when the scale is first administered.

The construct validity of the ROF-Ar was supported by strong convergent associations with HR and perceived exertion during graded exercise. As expected in an incremental treadmill protocol, perceptual and physiological indicators increased in parallel with workload [8, 10, 11]. Very high correlations during exercise have also been reported in the original ROF validation and in later adaptations and are generally interpreted as reflecting synchronized responses to increasing task demands rather than construct redundancy [8, 10]. Thus, the present findings are fully consistent with prior evidence regarding ROF behavior during progressive exercise.

The recovery phase provided the most discriminating test of construct specificity. During the 10-min passive seated recovery, the association between perceived fatigue (ROF-Ar) and perceived exertion (CR10) attenuated substantially, whereas moderate associations with HR persisted. Descriptively, perceived exertion and HR declined rapidly after exercise cessation, whereas perceived fatigue decreased more gradually and demonstrated greater interindividual variability. This attenuation is consistent with the intended discriminant behavior of the ROF during recovery, although it may also have been accentuated by reduced range and lower variability in CR10 ratings once exercise had ceased. This dissociation closely aligns with the recovery-phase behavior reported in the original ROF study and in the French [10] and Portuguese versions [11], in which perceived fatigue showed slower recovery kinetics than perceived exertion [8, 10, 11]. The replication of this pattern across linguistic versions provides supportive evidence for the discriminant validity and conceptual stability of the ROF during recovery.

From a comparative perspective, the ROF-Ar adds to the growing body of cross-cultural ROF validations. Although previous French [10] and Portuguese [11] adaptations followed broadly similar methodological principles, the contribution of the present study is not limited to procedural replication. Rather, it provides the first standardized Arabic version of the ROF and demonstrates that the distinction between perceived fatigue and perceived exertion is preserved in MSA during both graded exercise and recovery. This is particularly relevant in applied exercise, rehabilitation, and recovery-monitoring settings, where a specific measure of perceived fatigue may provide information that is not captured by exertion-based or physiological markers alone. Overall, the ROF-Ar extends the cross-cultural evidence base for the ROF by demonstrating that its intended construct structure and exercise–recovery behavior are reproducible in a major linguistic context that had not previously been examined.

It should be acknowledged that a Kannada version of the ROF has been reported in a clinical endodontic setting [23], suggesting potential applicability beyond sport and exercise. However, direct comparison with the present findings is limited, as that study did not employ a comparable exercise-and-recovery validation framework. Accordingly, the present results extend the evidence base for the ROF primarily within exercise and recovery contexts, where the conceptual distinction between fatigue and exertion is particularly relevant.

From a theoretical perspective, these findings are consistent with models that propose distinct temporal dynamics for cardiovascular recovery, effort perception, and the subjective experience of fatigue [1, 2, 9]. The ability of the ROF-Ar to reproduce the exercise‒recovery dissociation observed in previous studies reinforces the interpretation of perceived fatigue as a construct that is related to, but not reducible to, exertion or physiological strain.

From an applied perspective, the present results reinforce recommendations emerging from earlier ROF validation studies regarding the complementary use of perceptual and physiological indicators. During exercise, ROF-Ar can be used alongside HR and CR10 to characterize internal load and perceived strain [6]. During recovery, however, ROF-Ar provides specific and nonredundant information on perceptual recovery that is not captured by exertion scales or HR normalization alone. This added value during recovery has been observed consistently across the original ROF and multiple language versions, strengthening confidence in its practical utility for monitoring training load, recovery status, and readiness to perform in both athletic and clinical populations [5, 7, 8, 10, 11].

## Limitations

Several limitations should be acknowledged. First, the present study did not assess test–retest reliability, measurement error, or responsiveness to change. This was a deliberate design choice, as the study was intended as an initial cross-cultural adaptation and validity assessment, consistent with previous ROF translation studies. Notably, similar staged validation approaches were adopted in the French and Portuguese ROF adaptations, with reliability and responsiveness examined in subsequent work [10, 11].

Second, a substantial proportion of the face-validity sample was drawn from sport- and exercise-related professions, which may have facilitated the distinction between perceived fatigue and perceived exertion. Although this composition was useful for an initial applied validation, it may limit generalizability to broader Arabic-speaking populations with less familiarity with perceptual monitoring concepts. In addition, prior exposure to the original ROF, other translated versions, or the conceptual distinction between fatigue and exertion was not systematically assessed and may have influenced some responses.

Third, perceived exertion was assessed using the French version of the Borg CR10, because no standardized and validated Arabic version was available at the time of testing. Nevertheless, all participants had received academic education, and French is widely used in Tunisia from the early years of schooling through higher education, which likely reduced the risk of misunderstanding. However, understanding the French Borg CR10 does not necessarily imply equivalent comprehension of more conceptually nuanced perceptual instruments such as the ROF. The CR10 is relatively brief and familiar in Tunisian sport and exercise settings, whereas the ROF requires respondents to interpret fatigue-specific descriptors and distinguish perceived fatigue from perceived exertion. This point should nonetheless be considered when interpreting the findings.

Fourth, construct validity was examined using a treadmill-based incremental protocol in healthy adults. Although this controlled context was appropriate for initial validation and facilitated direct comparison with previous ROF studies, generalization to other exercise modalities (e.g., intermittent or resistance exercise) and to clinical or athletic populations requires further investigation.

Finally, the magnitude of correlations observed during graded exercise was partly attributable to the ordered, time-dependent nature of the protocol. Accordingly, recovery-phase analyses should be considered the primary source of evidence for discriminant validity rather than exercise-phase correlations alone.

## Conclusion

The ROF in Modern Standard Arabic (ROF-Ar) demonstrated high semantic and conceptual equivalence with the original version, adequate face validity, and supportive evidence of construct validity. Its pattern of association during graded exercise, together with its dissociation from perceived exertion during post-exercise recovery, supports the intended conceptual distinction between perceived fatigue and perceived exertion. Overall, ROF-Ar represents a brief, clear, and practical tool for assessing perceived fatigue in Arabic-speaking adults across exercise and recovery contexts. Future studies should further examine the reliability, responsiveness, and broader applicability of the ROF-Ar across diverse Arabic-speaking populations, exercise modalities, and real-world settings, including longitudinal or intervention-based designs assessing training-induced fatigue and recovery dynamics.

## Supplementary Information

Additional file1 (DOCX 17 KB)

## Data Availability

Data associated with this paper can be produced on request from the corresponding author.
