# Supplementary material for: Cross-Cultural Adaptation and Validation of the Arabic Rating-of-Fatigue Scale
Source: Sports Med Open. 2026 Jun 19;12:76. doi: 10.1186/s40798-026-01052-7 (PMC13282412; doi:10.1186/s40798-026-01052-7)
Supplement: Supplementary file 1 — Additional file1 (DOCX 17 KB) [file 40798_2026_1052_MOESM1_ESM.docx]

Article title: Cross-cultural adaptation and validation of the Arabic Rating-of-Fatigue Scale
Journal: Sports Medicine – Open

Authors: Mohamed Ali Baccouche, Khaled Trabelsi, Liwa Masmoudi, Haitham Jahrami, Achraf Ammar, Hamdi Chtourou
Corresponding author: Dr. Achraf Ammar
Affiliation: Department of Training and Movement Science, Institute of Sport Science, Johannes Gutenberg-University Mainz, Mainz, Germany
E-mail: acammar@uni-mainz.de

**Figure S1. Arabic version of the Rating-of-Fatigue Scale (ROF-Ar).**

**مقياس تقييم التعب**
**النسخة العربية**
**(ROF-Ar)**

يرجى تقييم مدى شعورك بالتعب الآن (هنا والآن).

يشير الرقم 0 إلى عدم الشعور بالتعب إطلاقًا، ويشير الرقم 10 إلى تعبٍ كامل وإرهاقٍ تامّ (لا طاقة متبقّية).

**ملاحظة:** يهدف هذا المقياس إلى تقييم الشعور بالتعب وليس شدة الجهد المبذول.

| **الرقم** | **الوصف اللفظي** |
| --- | --- |
| 10 | تعب كامل وإرهاق تام: لا طاقة متبقّية |
| 9 |  |
| 8 | متعب جدًا |
| 7 |  |
| 6 |  |
| 5 | متعب بشكل معتدل |
| 4 |  |
| 3 | متعب قليلًا |
| 2 |  |
| 1 |  |
| 0 | غير متعب على الإطلاق |
